# The Norwegian Healthy Life Study: protocol for a pragmatic RCT with longitudinal follow-up on physical activity and diet for adults

**DOI:** 10.1186/s12889-016-3981-1

**Published:** 2017-01-05

**Authors:** Eirik Abildsnes, Eivind Meland, Thomas Mildestvedt, Tonje H. Stea, Sveinung Berntsen, Gro Beate Samdal

**Affiliations:** 1 Department of Global Public Health and Primary Care, University of Bergen, Bergen, Norway; 2 Department of Public Health, Sport and Nutrition, University of Agder, Kristiansand, Norway; 3 Department for research and development, Haukeland University Hospital, Bergen, Norway

**Keywords:** Randomized control trial, Health behaviour, Physical activity, Diet, Adults

## Abstract

**Background:**

The Norwegian Directorate of Health recommends that Healthy Life Centres (HLCs) be established in primary health care to support behaviour change and reduce the risk of non-communicable diseases. The aim of the present study protocol is to present the rationale, design and methods of a combined pragmatic randomized controlled trial (RCT) and longitudinal cohort study of the effects of attending HLCs concerning physical activity, sedentary behaviour and diet and to explore how psychological well-being and motivational factors may mediate short— and long-term effects.

**Methods:**

The present study will combine a 6-month RCT with a longitudinal cohort study (24 months from baseline) conducted at six HLCs from June 2014 to Sept 2017. Participants are randomized to behavioural change interventions or a 6-month waiting list control group.

**Discussion:**

A randomized trial of interventions in HLCs has the potential to influence the development of policy and practice for behaviour change interventions and patient education programmes in Norway. We discuss some of the important preconditions for obtaining valid results from a complex intervention and outline some of the characteristics of ecological approaches in health care research that can enable a pragmatic intervention study.

**Trial registration:**

The study was retrospectively registered on September 19, 2014 and is available online at ClinicalTrials.gov (ID: NCT02247219).

## Background

Lifestyle risk factors are recognized as a leading contributor to morbidity and mortality in Europe due to the development of non-communicable diseases (NCDs). There is by now solid evidence for the causal link between regular physical activity (PA), healthy dietary habits and good health [1]. The WHO’s Global Action Plan urges national governments to develop NCD targets and plan how the health care system should respond to these targets [1]. As part of the national NCD strategy [2], the Norwegian Directorate of Health recommends that Healthy Life Centres (HLCs) be established in primary health care [3]. The target group is persons of all ages with a high risk of contracting a disease, or who are already living with a disease and need help to change their health behaviour and manage their condition.

HLCs offer individual and group-based behavioural change intervention programmes focusing mainly on the promotion of healthy dietary and physical activity habits as well as smoking cessation. At a system level, HLCs aim to function as a resource, knowledge and contact centre for behaviour change, health promotion and disease prevention in the municipalities. By targeting NCD risk in vulnerable groups, HLCs are one of the national strategies and efforts aiming to reduce social health inequalities [4]. By the end of 2014, 57% of Norwegian municipalities provided HLC activities, and the number of established HLCs doubled during the period 2011–2014 [5].

However, the scientific evidence for health promotion effectiveness is not convincing in a primary care setting similar to HLCs [6], and the pathways and mediators linking unhealthy behaviour to deteriorated health are not well understood [7]. A review study evaluating the effectiveness of interventions comparable to the Norwegian HLC model reported conflicting results, noting that the included studies were hampered by methodological insufficiencies [8].

Behavioural change intervention programmes at HLCs are complex interventions, with a number of interacting components and outcomes. In complex interventions based on real-life settings, randomized controlled trials (RCTs) may have limited impact on practice and policy, since the impact of interacting contextual factors differs by location [9]. Lewis et al. suggested to design theory-based interventions and include theory-derived mediating variables to identify effective interventions and techniques [10]. The UK Medical Research Council (MRC) has developed guidance to design and evaluate complex interventions [9]. A realist evaluation approach may enable complex interventions to address questions about what works, for whom and under what circumstances [11], and take into account that generation of knowledge may come from practitioners involved in a study as well as from the researchers [12].

The Norwegian Directorate of Health recommends that HLCs adopt an approach based on salutogenesis [13], and use motivational interviewing (MI) as a counselling approach [14]. The trans-theoretical model of change [15], used in addition to MI, provides counsellors with a conceptual model to explain why some people change while others do not [16]. Self-determination theory (SDT) suggests that counsellors may enhance behaviour change and maintenance of new habits by positively influencing the quality of clients’ motivation by supporting the three basic psychological needs, namely autonomy, competence and relatedness [17]. Need-supportive interventions and a more autonomous regulation of behaviour have been shown to predict success in many domains, including long-term weight control [18], tobacco dependence [19], predicting psychological well-being [20] and exercise [21]. Moreover, successful self-regulation in physical activity has been shown to spread and affect other behaviour domains, such as the regulation of eating [22]. Autonomous regulation of eating has been associated with healthier eating, being concerned with what one eats (the quality of food), a predictable reduction in food calories, eating more fruits and vegetables and food planning [23]. Body dissatisfaction, obesity and dysfunctional eating are often associated with a controlled regulation of eating behaviour [24]. Even though MI has been developed as a clinical tool and SDT is an empirically based theory, there are similarities and conceptual overlap between them [25]. MI supports the participants’ need for autonomy and relatedness by allowing them freedom to explore reasons for and against change (autonomy) in a non-judgemental context (relatedness) [25].

The HLC model is still in development, and is expected to expand and include patient education and self-management programmes targeting the most prevalent NCDs [3]. Consequently, there is a lack of studies evaluating the effect of HLC programmes. Results from a prospective intervention study with a 12-month follow-up indicated that participation in a group-based prescribed PA programme for 3 months significantly improved physical fitness and health-related quality of life (HRQoL) post intervention and at follow-up [26]. However, the generalizability of these findings is affected by high drop-out rates and should therefore be interpreted with caution. A qualitative study by Følling et al. [27] indicated that emotional distress among Norwegian HLC participants may hamper behaviour change; doubts were raised about whether HLC interventions are sufficient to provide maintenance of change due to previous negative life experiences, shame and low self-efficacy among the participants. Thus, there is a need to evaluate the effects of the Norwegian HLC model.

In the process of developing the intervention study described in this protocol paper, we have previously reported a focus group study exploring stakeholders’ expectations at seven different HLCs in small and large municipalities [28]. We explored the local adaptation of the HLC model and the contextual diversity of behavioural change programmes and competence available at different sites. Based on this understanding, we designed an RCT based on common intervention components, methods and theoretical input at the HLCs included in the study.

### Aims

The aims of the present study were to evaluate (1) the short— and long-term effects of behavioural change intervention in Norwegian HLCs on physical activity, self-perceived health and well-being, self-reported diet and eating behaviour, tobacco use, and sleep and body concern, (2) the factors that mediate these effects and (3) the possible adverse effects of the intervention.

## Methods/design

The Norwegian Healthy Life Study is a 6-month RCT with a longitudinal follow-up (24 months after inclusion) to assess the effectiveness of behaviour change interventions in HLCs for adults, with the underlying purpose being to develop a pragmatic intervention informed by an ecological model of health [29]. Based on theoretical assumptions and previous research, we hypothesize that (1) an increase in PA and a healthier diet will be observed in the intervention group, compared with the waiting list control group, (2) participants who experience the health personnel as supportive of autonomy will report more autonomous reasons, less nudging and less psychological defiance of behaviour change during short— and long-term follow-up and (3) beneficial changes in motivation and well-being will ameliorate socio-economic differences in maintenance of behaviour change at follow-up.

The study will be reported in accordance with the Consolidated Standards of Reporting Trials (CONSORT) statement [30] and the Template for Intervention Description and Replication (TIDieR) [31]. The protocol is available online at ClinicalTrials.gov (ID: NCT02247219).

### Setting

The members of the research group invited 12 municipalities to participate in the research programme. Four declined (one due to other research commitments at the HLC), leaving a sample of eight municipalities (with 6,000–270,000 inhabitants) with a total number of 630,000 inhabitants living in rural and urban areas on the west and south coast of Norway. The study is designed as a pragmatic RCT, based on an ecological understanding that behaviour change interventions must take into account the participants’ personal aspects (microsystem), their close supporters (mesosystem), the everyday environmental factors (exosystem) and finally structures and regulations on a systems level (macrosystem) [29].

Throughout the development of the intervention, we studied the HLC activities in different settings, and arranged seminars with involved counsellors, leaders and representatives of patient organizations [28]. Themes at these seminars included discussions of MI counselling, relevant theory, experiences in face-to-face counselling in a HLC setting, social determinants of health, aspects related to behaviour change among immigrants, medical ethics, dietary counselling and how to improve PA. In line with current recommendations for pragmatic RCTs, the local implementers were treated as co-learners in the development of the intervention model. The meetings with implementation staff built on local experiences and emphasized existing competence and skills. In meeting with the different local professional groups, the research group conveyed interest in individual and organizational challenges and emphasized support and respect for local competence and the quality of services.

### Inclusion criteria

Patients had to be ≥18 years old and able to participate in a group intervention held in the Norwegian language.

### Exclusion criteria

These included having disabling mental illness, mental retardation or only attending a smoking cessation intervention and not a PA and/or diet intervention.

### Recruitment

The local HLCs invited 351 persons (59% women) to participate in the study. Participants were referred by their general practitioner (GP), other health professionals or initiated attendance themselves. In the period June 2014–September 2015, 118 participants (34% of those invited; 77% women) were recruited. The main reason for refusing participation was the possibility of having to wait 6 months for the intervention if randomized to the waiting list control group.

### Interventions

**Fig. 1:**
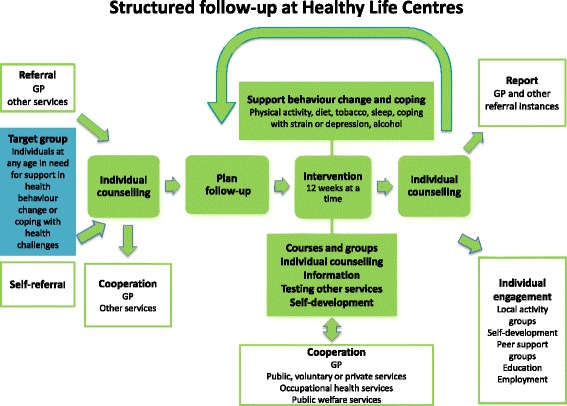
The Norwegian Healthy Life Centre model

The intervention group receives interventions according to the Norwegian Healthy Life model, as defined by the Norwegian Directorate of Health [3]. The model consists of (1) an individual counselling session based on referral from a GP, other health care providers or self-referral, (2) group-based behavioural change interventions for 12 weeks and (3) an individual counselling session by the end of the intervention (Fig. 1). The counselling sessions are based on MI.

The organization of the HLCs and the content of the intervention vary between the municipalities according to local resources and competence. Depending on availability, professionals involved may be physiotherapists, nutritionists, occupational therapists, trained lifestyle counsellors and PA instructors with a bachelor’s or master’s degree in nutrition and/or sports science and health promotion; or nurses trained in public health or psychiatry. During the first individual session of 30–60 minutes, the counsellor elicits and acknowledges the participant’s perspective on health, offers information about health consequences, and outlines the HLC’s PA, diet and/or stop smoking intervention support. Strategies are discussed to overcome barriers and facilitate change and set realistic targets. Graded goals for behaviour change are negotiated and confirmed in a written action plan.

The participants are encouraged to monitor their behaviour, e.g. in a log-book, and use web-based applications for support, e.g. the national stop smoking app. Group-based PA consisting of elements from aerobic training (e.g. Nordic walking), light strength training, stretching and games is encouraged twice a week. A course promoting healthy eating (10 hours) includes information about meal composition, beverages, meal size, and demonstration and practice, e g. how to read food labels and prepare healthy food and beverages. If intending to stop smoking, participants are offered group-based smoking cessation counselling. The group-based interventions provide opportunities for social support and encouragement among participants in the same situation.

After 12 weeks of participation in group-based activities, there is a second individual counselling session of 30–60 minutes to review behaviour goals and the outcomes of behaviour change, e.g. weight loss and fitness, with the counsellor offering feedback on results. If there is a need and motivation for further interventions, the participants may extend their participation period several times, up to one year. Some HLCs ask for a small fee (ca. €50) for attending the HLC programme to increase the participant’s commitment to the programme. After the intervention (at 6 months) and at the 24-month follow-up, the participants are asked about the types of intervention they attended and how long their participation lasted.

### Control group

The control group receives the same intervention after a waiting period of at least 6 months. The control group was told to live as normal, and no restriction was given with respect to behaviour change. The majority of the HLCs included new participants according to local capacity, with the consequence that both intervention and control group participants may have to wait for a while.

### Randomization and allocation

Participants are randomly assigned by a simple method using a random number list and an approach that ensures equal distribution in the intervention and control groups. A project co-ordinator, working outside the HLC premises, assigns participants to either the intervention group or the waiting list by drawing cards from numbered sealed 2envelopes after the inclusion visit and registration of inclusion data, thereby ensuring concealment of the sequence to those enrolling the patients and of the identity and patient characteristics to the researcher. A block randomization is performed with randomization stratified by trial site in blocks of 20 to avoid uneven distribution of participants at any of the HLCs.

### Blinding

It is not possible to blind either the participants or the staff performing the interventions to group allocation. Blinding of assessment is aimed at by means of objective PA and sedentary time measurements (described below) and by online self-reported data collection (described below).

### Data collection

Self-reported data are collected by an online system for survey management, SurveyXact™ (Rambøll Management Consulting, Oslo, Norway). The counsellors help the participants to access the online survey, and are then left alone in a separate room until the survey is completed. The survey was tested on four participants at two HLCs, who found the questions understandable and possible to complete in 30–45 minutes. Data are collected at the local HLC prior to randomization (baseline), after 6 months (post intervention) and 24 months after baseline from the intervention group participants. Waiting list controls perform registration of data at inclusion, after 6 months on the waiting list, at 12 months (post intervention) and at the 24-month follow-up. A SPIRIT flow diagram illustrates the data collection in the intervention group and control group [32] (Tables 1 and 2).

**Table 1:** The intervention group

Intervention group	Study period			
	Enrolment	Allocation		
	T_0_		T_2_ 6 months	T_4_ 24 months
Enrolment				
Eligibility screen	x			
Informed consent	x			
Allocation		x		
Intervention				
Assessments				
Biomedical data	x		x	x
Socio-demographic data	x			
PA monitor	x		x	x
PA questionnaire	x		x	x
Self-perceived health and well-being	x		x	x
Diet and eating behaviour	x		x	x
Tobacco use	x		x	x
Sleep	x		x	x
Body concern	x		x	x
Social support	x		x	x
Defiance	x		x	
Regulation of motivation	x		x	x
Perceived autonomy support			x	
Self-efficacy for PA	x		x

**Table 2:** The control group

Control group	Study period				
	Enrolment	Allocation	Post allocation		
	T_0_		T_1_ 6 months	T_3_ 12 months	T_4_ 24 months
Enrolment					
Eligibility screen	x				
Informed consent	x				
Allocation		x			
Intervention					
Assessments					
Bio-medical data	x		x	x	x
Socio-demographic data	x				
PA monitor	x		x	x	x
PA questionnaire	x		x	x	
Self-perceived health and well-being	x		x	x	x
Diet and eating behaviour	x		x	x	x
Tobacco use	x		x	x	x
Sleep	x		x	x	x
Body concern	x		x	x	x
Social support	x		x	x	x
Defiance	x		x	x	
Regulation of motivation	x		x	x	x
Perceived autonomy support				x	
Self-efficacy for PA	x		x	x	

### Biomedical and socio-demographic data

At inclusion, the counsellors at the HLCs measure the participant’s weight, height and waist circumference (light clothing, no shoes), and give each participant a unique number in the survey. Waist circumference is measured at the level of the umbilicus. The questionnaire includes questions about socio-demographic data, the reasons for attending the HLCs, and total time of participation and types of intervention received at follow-up.

### Primary outcomes

Primary outcome measures will be the objective measurement of moderate-to-vigorous physical activity (MVPA).

#### Physical activity

Participant’s PA will be recorded (1) objectively by a PA monitor (SenseWear™ Armband Mini, BodyMedia Inc., Pittsburgh, PA, USA) and (2) by two survey questions: “In general, for how long are you physically active each day?”; and “How hard do you exercise?”. These questions have been previously validated in comparison with biological markers in Norwegian adults [33]. Study participants are instructed to wear the monitor on the upper left arm (the triceps muscle), according to the manufacturer’s instructions, for 24 hours a day for 7 consecutive days, except for water-based activities.

The monitor is reliable, valid and suitable for measuring daily living PA in normal and overweight adults [34, 35]. Data are downloaded with the manufacturer’s software (SenseWear™ Professional Research Software Version 7.1, BodyMedia Inc). The analysis includes only data from participants with ≥4 valid days of measurements. Valid data should cover at least 19.2 hours during that given day, i.e. 80% of a 24-hour sampling period. PA intensity is defined using metabolic equivalents of task (METs) as minutes spent sedentary (≤1.0–1.4 METs), light PA (1.5–2.9 METs) and MVPA (≥3 METs). Thus, sedentary time, steps per day and light PA are used as secondary outcomes.

### Secondary outcomes

Secondary outcome variables will also include self-perceived health and well-being, self-reported diet and eating behaviour, tobacco use, sleep and body concern.

#### Self-perceived health and well-being

Self-rated health is measured by the single item question “How is your overall health at the moment?” previously used in a Norwegian study [36]. The four response categories are “Very good”, “Good”, “Not so good” and “Poor”.

Information on quality of life is assessed using Cantril’s ladder [37]. The Impact of Weight on Quality of Life-Lite Questionnaire (IWQOL-Lite) is a validated, self-report measure of obesity-specific quality of life [38]. In this study, we use nine of the 31 items that cover quality of life in relation to the domains physical function and self-esteem.

The single-item self-esteem scale (SISE) is used to assess global self-esteem [39]. The IWQOL-Lite also contains a self-esteem construct with four items [38]. The scales have proved to have strong construct validity when applied to adult populations.

Vitality is assessed by the Subjective Vitality Scale, a measure of the state of feeling alive and alert, and of having energy available to the self [40]. Vitality is considered an aspect of eudemonic well-being [41].

In studies linking childhood experience of parental acceptance and rejection to adult behavioural and emotional adjustment, the phenomenological perspective, i.e. the remembrance and the personal evaluation of the relation with caregivers, is the most prominent [42]. We have included a single self-assessment item of the quality of childhood, similar to a question that proved to be associated with multi-morbidity and allostatic load in a recent Norwegian study [43].

#### Diet and eating behaviour

The survey includes questions on meal pattern, and habitual diet and beverage consumption. The questions assessing meal frequency, meal composition and use of beverages were previously used in Norwegian health surveys [44]. Meal frequency is assessed by questions such as “How often do you have breakfast each week?” with the same asked for lunch, dinner and supper. Response alternatives range from never or seldom to seven days a week.

Beverage consumption is assessed by questions such as “How often do you drink water, regular soft drinks, diet soft drinks, lemonade and fruit juice?”; consumption of food items is assessed by questions such as “How often do you eat candy, salty snacks, cakes/cookies/pastries, fast food, nuts, high-fat and low-fat dairy products, fish, red and white meat and oils?”. The frequency of food and beverage consumption is assessed by ticking response alternatives coded per week or per day.

We emphasize diet items pertaining to the Mediterranean diet because this diet has documentation on hard end-points in secondary as well as in primary preventive studies [45, 46]. The Mediterranean diet index includes 11 main components of the Mediterranean diet (unrefined cereals, fruits, vegetables, potatoes, legumes, olive oil, fish, red meat, poultry, full-fat dairy products and alcohol) [47].

The Three-Factor Eating Questionnaire-R18 is an 18-item questionnaire previously used in an intervention study targeting obese subjects in Norway [48], and is considered a robust scale to measure cognitive restraint, uncontrolled eating and emotional eating.

#### Tobacco

Use of tobacco will be assessed by the single question “Do you smoke or use snuff?” with “Yes, I smoke daily”, “Yes, I smoke but not daily”, “Yes, I use snuff daily”, “Yes, I snuff but not daily” or “No” as alternative responses.

#### Sleep

A structured log-book with five items assesses sleep patterns [49]. The participants are instructed to write a report first thing on seven consecutive mornings.

#### Body concern

We use three questions pertaining to body concern validated in the Health Behaviour in School-aged Children study [50] and the Body Attitude Test with seven items measuring lack of familiarity with one’s body [51].

### Mediating variables

Mediating variables include social support in general, social support for PA, defiance, regulation of motivation, perceived autonomy support and self-efficacy for PA.

#### Social support in general

The Oslo-3 Social Support Scale (OSS-3) with three questions, previously used in Norwegian context, assesses social support [52, 53]. A mean score is estimated from a minimum of two questions.

#### Social support for PA

Social support for PA from friends and family is measured using a scale developed by Sallis et al. [54] previously used in Norwegian surveys [55].

#### Defiance

Psychological defiance pertains to the tendency of oppositional rejection of advice and opinions from authority persons. Four items derived from research on parenting styles with a high inter-item reliability were adapted and slightly changed in wording to fit our context [56, 57].

#### Regulation of motivation

The Treatment Self-Regulation Questionnaire (TSRQ) (15 items) assesses the degree to which a person’s motivation for a particular behaviour is relatively autonomous or self-determined. In this case, the particular behaviour is joining a behaviour change programme and following its guidelines for exercise and a healthy diet, or continuing to follow the guidelines after the programme has ended. The questionnaire was validated by Levesque et al. [58] and has been used in various studies, including in Norway [59]. The scale identifies differences in types of regulation (subscales), amotivation (lacking any intention to engage in behaviour) (3 items), and controlled (6 items) and autonomous motivation for behaviour change (6 items). Responses are rated on a 7-point Likert scale ranging from “strongly disagree” to “strongly agree”. Examples of items included in the different subscales are “I really don’t think about it”, “Because I want others to see that I can do it”, and “Because I feel that I want to take responsibility for my own health”. The subscales are averaged and can be used separately.

#### Perceived autonomy support

The 6-item version of the Health Care Climate Questionnaire (HCCQ) measures the degree to which patients experience their health care providers to be autonomy supportive versus controlling in counselling with respect to behavioural change [19]. Responses are rated on a 7-point Likert scale ranging from “strongly disagree” to “strongly agree”. Higher scores represent greater perceived support for autonomy by health care professionals after an intervention. This instrument has been extensively validated and used in various studies targeting obesity, smoking cessation, diet improvement and regular exercise [60, 61], also in a Norwegian setting [62]. The HCCQ was reduced from six to four items in the present study after tests on a dataset of patients with coronary artery disease showing no loss of inter-item reliability (Cronbach’s alpha = 0.89). Due to ceiling effects and low variability in a former study [63], the midpoint on the scale was moved in the opposite direction of the ceiling, yielding acceptable variability in each of the four items and with absolute values of skewness <1.0.

#### Self-efficacy for PA

Self-efficacy for PA is assessed by a questionnaire previously used in Norwegian studies [44, 64], representing eight psychological and five practical barriers. Participants are asked to indicate on a 7-point Likert scale (ranging from “not at all confident” to “extremely confident”) to what extent they were confident in their ability to perform planned PA in the face of potential barriers.

### Sample size and statistical power

Power calculations showed that 51 adults are required in each group to obtain 80% statistical power with a 5% significance level, and to detect a between-group difference in MVPA of 10 (standard deviation 20) min/day. To account for drop-out, 118 persons are included, 57 in the intervention group and 61 in the control group.

### Statistical analysis

Data are presented by descriptive statistics. Statistical analysis is conducted by SPSS (Statistical Package for the Social Sciences) or equivalent. The study provides standard means and deviation of each variable for the participants in the intervention and control groups. The waiting list design controls for cross-over and interaction effects. We also perform intention-to-treat analyses with conservative estimates of missing data. A baseline comparability analysis across the two intervention groups is also carried out, with results expressed by means and ± standard deviation. To compare means, analysis of variance or *t* tests are performed; Mann–Whitney *U* tests are used to compare variables with non-normal distribution. Intervention effects are evaluated performing general linear modelling. Mediator and moderator analyses will apply regression analyses.

## Discussion

The MRC guidance on developing and evaluating complex interventions puts emphasis on theoretical understanding of how the intervention causes change, identification of implementation problems, consideration of sample size based on variability of individual-level outcomes due to higher-level processes, a range of measures instead of a single outcome, and a specified degree of adaptations to local context instead of strict standardization [9]. In the present study, we have selected multiple measures informed by theories of behavioural change with SDT as a point of departure in an ecological approach [29]. SDT supports an ecological understanding of behaviour where no priority is placed on the individual, group or environment. Relatedness is built when the client feels understood, cared for and valued by significant others (family, health personnel, community). This also emphasizes how the social context may support or thwart optimal motivation [17].

Context is important in research on health behaviour change, and knowledge translation, practice implementation and health improvement are dependent on local factors. Many intervention and evaluation designs seek to eliminate contextual confounders. In opposition to this view, we maintain that contextual factors represent the normal conditions into which interventions must be integrated if they are to be workable in practice [65]. In the present study, a strategic sample of municipalities representing diverse contexts participates, with the aim of increasing the external validity of the study.

### Strengths and limitations

A pragmatic approach taking into account local resources and preferences should enhance the external validity of our findings. On the other hand, the intervention is not optimally standardized. However, the call for standardizing complex interventions is a “double-edged sword” often leading to a lack of local ownership and low quality of the interventions and even sapping the effectiveness of well-designed studies [66]. The consequence of this concern is not to abandon RCTs in health services research, but rather to emphasize process and not content standardization. With an emphasis on process, we may develop interventions that are sensitive to local contexts with a focus on promoting competence, and safeguarding local ownership and autonomous motivation also for the providers [67, 68].

The waiting list group design has some obvious weaknesses, e.g. measuring compliance to waiting as well as the effect of the intervention. Only 35% of those invited accepted to take part in the study, which might weaken the external validity of the study. If we experience unbalanced drop-out with a high attrition rate in the waiting list group, the internal validity of the study will obviously be affected. We have accounted for drop-out and have reached the number of participants recommended based on power calculations. The primary outcome measure will be objectively assessed, and validated tools will assess secondary and mediating variables. The research team possesses competencies in sports and nutrition sciences, general practice, nursing sciences and public health.

The results may also be relevant for other countries with comparable health care systems in the search for effective interventions for NCD targets.

## Conclusion

A randomized trial of interventions in Healthy Life Centres has the potential to influence the development of policy and practice for behaviour change interventions and patient education programmes in Norway.

## Abbreviations

CBT: Cognitive behavioural therapy
GP: General practitioner
HCCQ: Health care climate questionnaire
HLC: Healthy life centre
HRQoL: Health-related quality of life
METs: Metabolic equivalent of task
MI: Motivational interviewing
MRC: Medical research council
MVPA: Moderate-to-vigorous physical activity
NCD: Non-communicable disease
PA: Physical activity
RCT: Randomized controlled trial
SDT: Self-determination theory
TSRQ: Treatment self-regulation questionnaire
